# Vision-Based Georeferencing of GPR in Urban Areas

**DOI:** 10.3390/s16010132

**Published:** 2016-01-21

**Authors:** Riccardo Barzaghi, Noemi Emanuela Cazzaniga, Diana Pagliari, Livio Pinto

**Affiliations:** Department of Civil and Environmental Engineering (DICA)-Geodesy and Geomatics Section, Politecnico di Milano, Piazza Leonardo da Vinci 32, Milan 20133, Italy; riccardo.barzaghi@polimi.it (R.B.); diana.pagliari@polimi.it (D.P.); livio.pinto@polimi.it (L.P.)

**Keywords:** global positioning system, ground penetrating radar, image processing, photogrammetric positioning

## Abstract

Ground Penetrating Radar (GPR) surveying is widely used to gather accurate knowledge about the geometry and position of underground utilities. The sensor arrays need to be coupled to an accurate positioning system, like a geodetic-grade Global Navigation Satellite System (GNSS) device. However, in urban areas this approach is not always feasible because GNSS accuracy can be substantially degraded due to the presence of buildings, trees, tunnels, *etc*. In this work, a photogrammetric (vision-based) method for GPR georeferencing is presented. The method can be summarized in three main steps: tie point extraction from the images acquired during the survey, computation of approximate camera extrinsic parameters and finally a refinement of the parameter estimation using a rigorous implementation of the collinearity equations. A test under operational conditions is described, where accuracy of a few centimeters has been achieved. The results demonstrate that the solution was robust enough for recovering vehicle trajectories even in critical situations, such as poorly textured framed surfaces, short baselines, and low intersection angles.

## 1. Introduction

An accurate knowledge about the geometry and position of underground utilities is a fundamental aspect for their efficient management, optimal planning, and cheap maintenance with minimum effect on traffic and users. In this context, Politecnico di Milano has been involved in a research project studying a new system for 3D mapping of underground utilities and the development of an automatic laying system (trencher) for electrical cables. Localization of underground objects with the high level of accuracy required by trencher navigation is usually performed by *ad hoc* Ground Penetrating Radar (GPR) surveys ([1,2,3,4]) in order to avoid damage to buried cables during digging. GPR surveying [5] is based on the emission of electromagnetic pulses with frequencies between 10 and 2000 MHz and on their reflection due to underground discontinuities. This allows the evaluation of the depth and position of underground items and the identification of their main geometry. To allow the 3D description of identified objects, the GPR device is coupled to a suitable positioning system that determines its position with accuracy on the order of 0.2 to 0.3 m. In an open sky environment, the optimal approach for georeferencing is Global Navigation Satellite System (GNSS) positioning. It is fast, quite cheap and very accurate (on the order of 0.03 m or less, see [6]) when a phase differential solution is adopted. Nevertheless, this solution requires at least four satellites commonly visible to both the rover and the master antennas. In urban areas sky visibility is often partially obstructed and satellite signal leakages, poor satellite distribution or low signal-to-noise ratio are not infrequent. Furthermore, for improving the reliability of the estimated positions, the obtained GNSS double differences fixed solutions should be filtered considering the estimated standard deviations and Position Dilution of Precision (PDOP) values [7]. Hence, accurate GNSS positioning is not always available during urban GPR surveys and so an alternative method is required. Considering our purpose, the positioning requirements can be summarized as follows:

- accuracy has to be on the order of 0.2 to 0.3 m;
- a real time solution and the attitude of the carrier are not strictly required;
- the system must manage protracted GNSS failures and even the case of no GNSS at all, considering, e.g., an urban canyon environment and the low speed at which GPR surveys are generally performed (less than 20 km/h);
- the methods should take advantage of the fact that the survey is often performed by almost parallel strips for covering the whole roadway;
- estimated coordinates should be referred to a known (preferably global or national) reference frame;
- the system has to be cheap.

Alternative methods for GNSS-challenged environments have been extensively studied by the research community: Mautz [8] recently presented a quite comprehensive overview of indoor positioning technologies, organizing them into 13 macro-classes. One of the most used approaches for georeferencing GPR surveys is the adoption of a robotic total station, able to auto-track a target prism mounted on the carrier ([9,10,11]). However, while this technology is successful in open areas, with quite continuous inter-visibility between total station and prism, on town roads signal obstructions caused by car and pedestrian transit are frequent. Other positioning methods can use techniques either needing the installation of sensors only on the vehicle or requiring sensors and/or infrastructures in the surrounding area too. For the sake of simplicity we selected a method belonging to the first group. The most important macro-classes, that can achieve the target accuracy, are inertial navigation, vision-based positioning and laser scanner positioning. Inertial techniques use so-called proprioceptive sensors, which provide information on the internal states of the vehicle, while the others use exteroceptive sensors. Both types require some external information, at least to initialize their states. Inertial positioning is widely used, but instruments able to give the required accuracy are very expensive. Moreover, if no GNSS signal is available, the solution rapidly drifts as a function of time, and it is not able to exploit the parallelism of GPR survey strips. Instead, the other two approaches show a drift which is a function of the traveled distance, that in our case is more advantageous than a drift in time. Moreover, they can consider, through the use of suitable algorithms, the overlap of the surveyed scenes across different strips. Between those, we decided to adopt a vision-based approach, which at the moment is the cheapest implementation that provides the required accuracy.

Vision-based localization techniques have also been very broadly studied [12]. These techniques require external information in order to determine absolute scale and the position solution can drift when GNSS or Ground Control Points (GCPs) are not available. Forlani *et al*. [13] obtained, using two front cameras in a stereo configuration, a position drift up to 0.5 m after covering a distance of 300 m in urban areas. Different approaches have been proposed to increase the performance of vision-based positioning. Soloviev and Venable [14] proposed the integration of GPS carrier phase measurements to constrain the vision solution obtained with a monocular videocamera. This approach resulted in an accuracy of less than 0.10 m when three satellites are visible. However, their tests also integrated an odometer and low-cost Inertial Measurement Unit (IMU) too, thus increasing the number of sensors and system cost. There are many other papers, such as [15,16], which propose a system integrating a camera, undifferenced GNSS and a low-cost IMU, which generally achieve errors of a few meters. Kim *et al*. [17] suggested determining car positions using built-in sensors, together with a front-facing camera and a low cost GPS, with a root mean squared error (RMSE) on the order of 2 m. Thus, it is clear that external information about the observed scene is required, in order to provide positioning without the use of GPS observations. Fang *et al*. [18] proposed placing a downward-looking camera on a vehicle: positioning in this case is achieved by matching the images to a previously generated global reference texture map. Odometry data was also used and the results show accuracy on the order of 0.1 m; however the generation of the global texture map is expensive and time-consuming. Chausse and Chapuis [19] proposed a vision system, combined with a low cost GPS and a numeric map of the road surface markings. This solution obtained 0.5 m error in the direction perpendicular to the road axis, while the error in the direction of the road axis was of some meters. Mattern *et al*. [20] integrated a detailed digital map of the road surface markings, with a camera, an odometer and a low-cost GPS, and were able to obtain absolute errors of less than 1 m. Lothe *et al*. [21], instead, used a coarse 3D environmental model in a Simultaneous Localization And Mapping (SLAM) solution. The accuracy for a synthetic sequence was on the order of 0.1 m, while results of a world test were not quantified. Soheilian *et al*. [22] proposed an integrated 3D geodatabase of buildings, roads and visual landmarks (reconstructed by multiple view aerial images with 0.10–0.25 m ground sampling distance) for aiding GNSS/vision positioning. High resolution textured façades were required and the obtained accuracy was not quantified. Qu *et al*. [23] suggested constraining the vision solution with a 3D landmark database of traffic signs. Results achieve accuracy of less than 0.5 m when a road sign is visible every 50–100 m. The major limit of those last approaches is that detailed 3D databases are relatively uncommon. Hence such databases must be prepared *ad hoc* for the survey, leading to increased time and cost. Barzaghi *et al*. [24] proposed to extract a selection of significant points from an existing large-scale urban map where sparse 3D points are often available (e.g., building corners) and to use them as GCPs. In these simulations the cameras were orientated to look at the roadside, and this approach gave errors of few centimeters. In subsequent work [25], this method was applied in a real test where a single photogrammetric strip of a road was collected. Despite the poor geometric calibration and the low quality of the urban map, the errors were on the order of 0.3 m after 150 m. Lastly, Pagliari *et al*. [26] adopted a similar strategy on a closed ring, with two vehicle mounted cameras. The RMSE for the whole route was never greater than 0.3 m, even when the relative orientation between the two cameras is not constrained, using 52 GCPs on a 600 m total distance. However, the characteristics of the route are quite far from real GPR surveys and none of these methods exploited the fact that they are performed along parallel strips. The purpose of this paper is to delineate a photogrammetric positioning method, only rarely supported by few GPS positions or GCPs, able to match the previously listed requirements, even in suboptimal conditions for the inverse photogrammetric problem. A real test is presented with data collected by a GPR, a camera and a GPS device installed on a wood carrier. Data were then processed following three different approaches: a purely photogrammetric solution, a photogrammetric solution integrating few GPS positions at the beginning and at the end of each strip, and another solution using few GPS solutions randomly distributed along some of the strips. In the following sections, firstly a detailed step-by-step description of the proposed methodology is discussed. Then, the real test is described, results are discussed and some conclusions are given.

## 2. The Photogrammetric Data Processing 

The proposed method requires the use of one or more photogrammetric cameras and a GNSS device, to be mounted on the same vehicle that is carrying the GPR. It is important to use cameras with fixed focal length, to acquire high quality images and to minimize the distortions. It is also recommended to use full-frame cameras, which guarantee a wider view and more contrasted images, reducing at the same time the signal-to-noise ratio. If the master station is sufficiently close to the survey site (in the order of few km), a single frequency GNSS device could be adopted, while in the other cases a double frequency device has to be used. The GNSS antenna should have good multipath reduction and sub-centimeter (or better) phase center stability.

The proposed photogrammetric solution is quite complex, but it allows georeferencing the GPR even in situations that may be critical for purely vision-based methods, e.g., poor-textured framed surfaces, only coplanar elements visible in the images, repetitive elements, short baselines between consecutive frames, low intersection angles, *etc*. The method can be schematized with three main steps: tie points (TPs) extraction from the images acquired during the survey, computation of approximate camera extrinsic parameters (EPs) by a first bundle block adjustment (BBA) and, finally, EPs refinement using a rigorous implementation of the collinearity equations, with another BBA. 

**Figure 1:**
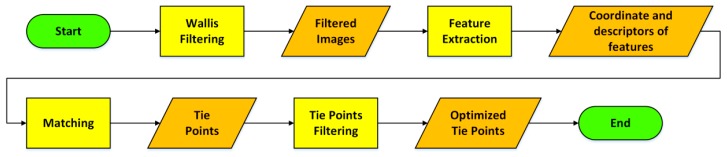
Methodological workflow describing tie point extraction.

**Figure 2:**
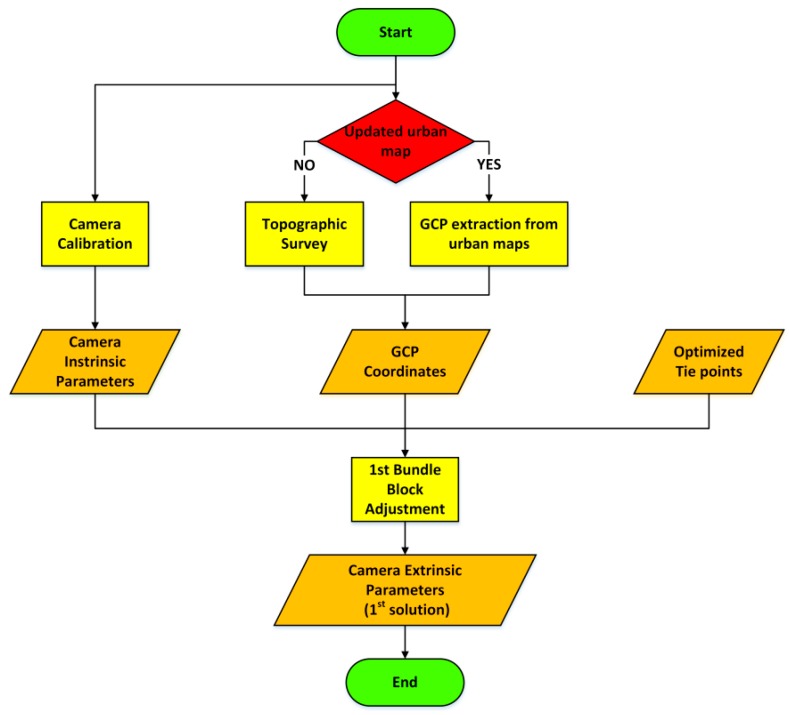
Methodological workflow of the first approximate bundle block adjustment.

The first step, TPs extraction (Figure 1), has been designed to obtain a good distribution of the TPs even in situations with bad textured surfaces. The use of automatic methods for TPs extraction is fundamental to increment the number of observations, ensuring at the same time a high accuracy (typically sub-pixel). Firstly, it can be advantageous to pre-process the images with the Wallis filter [27] to enhance the local contrast. After filtering, they are characterized by sharper details in both low and high contrast regions, that is crucial to ensure a good distribution of the tie points. These images are used as input for interest operators. They have to be able to automatically identify a high number of features in the urban environment that is characterized by a huge number of details. We tested several interest operators, based on different research approaches (corner detectors, regional interest operators *etc*.). It came out that multi-scale regional interest operators (such as SIFT [28] or SURF [29]) allows extracting a higher number of features, even in case of semi-uniformly textured surfaces. Moreover, the fact that these operators are scale independent allows one to better link together the different strips. In fact, the survey is performed following parallel strips, which is a requirement for GPR acquisitions, so the same scene is acquired at different scales. Then, the use of algorithms able to detect homologous points independently from the image scale is fundamental to link together the different strips of the photogrammetric block. The matching phase is performed using a multi-image approach, linking together overlapping images of different strips. This increases the TPs multiplicity and allows recovering the EPs under critical conditions. To speed up the computation, TPs reduction is performed sub-dividing the image space with a regular grid (whose spacing is user-defined). Within each cell, the point with highest multiplicity is selected and the corresponding observations on the other images are stored. We verified that the use of such strategy for TPs selection guarantees to maintain about 50 points for each image and reduces the BBA processing time, without loss of accuracy with respect to the use of all the observations. The computation of the BBA solution requires as input the camera intrinsic parameters (IPs), the image coordinates of the homologous points and a series of well distributed GCPs of known coordinates, necessary to constrain the degrees of freedom of the problem and to georeference the solution (Figure 2). The IPs and the lens distortions have to be determined performing a camera calibration prior to the survey. According to [30] IPs can be considered constant over the survey, however, they can be refined by performing a self-calibration and estimating the residual correction during the final BBA. The GCPs necessary to georeference the photogrammetric block must be well distributed along the survey and known with high precision. If these requirements are met it is not necessary to have a large number of GCPs. They can be measured, with a standard survey using a total station or extracted from urban maps, if the cartography of the surveyed area is available in a scale equal or larger than 1:1000. Of course the map has to be updated and the selected area must be properly represented. 

Starting from the optimized TPs, the GCPs coordinates and the IPs, a first approximate solution can be computed. The BBA is a non-linear problem (Equation (1)), so it requires approximate values to be solved:

(1) $$\{\begin{array}{c} x=x_{0}-c\frac{r_{11}(X-X_{0})+r_{12}(Y-Y_{0})+r_{13}(Z-Z_{0})}{r_{31}(X-X_{0})+r_{32}(Y-Y_{0})+r_{33}(Z-Z_{0})}\\ y=y_{0}-c\frac{r_{21}(X-X_{0})+r_{22}(Y-Y_{0})+r_{23}(Z-Z_{0})}{r_{31}(X-X_{0})+r_{32}(Y-Y_{0})+r_{33}(Z-Z_{0})} \end{array}$$

In Equation (1), c represents the principal distance, *x_0_* and *y_0_* represent the principal point offset, *X_0_*, *Y_0_* and *Z_0_* are the coordinates of the camera perspective centers, *X*, *Y* and *Z* the object coordinates and *r_ij_* are the elements of the 3 × 3 rotation matrix that depends on the gimbal angles *ω*, *ϕ*, and *κ*.

The proposed solution is computed in two steps: the first solution is just an approximate one, computed using the DLT (Direct Linear Transformation [31]) that implements a linear approximation of the photogrammetric triangulation problem, as shown in Equation (2):

(2) $$\{\begin{array}{c} x=\frac{L_{1}X+L_{2}Y+L_{3}Z+L_{4}}{L_{9}X+L_{10}Y+L_{11}Z+1}\\ y=\frac{L_{5}X+L_{6}Y+L_{7}Z+L_{8}}{L_{9}X+L_{10}Y+L_{11}Z+1} \end{array}$$

where x and y are the image coordinates, *X*, *Y* and *Z* are the object coordinates and *L_1,2…n_* are linear coefficients that are function of the IPs and EPs.

**Figure 3:**
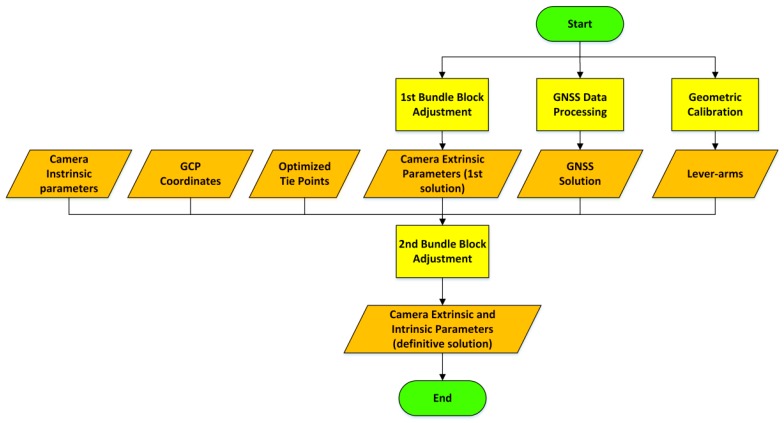
Methodological workflow for the rigorous bundle block adjustment.

The first step is required to generate the input data necessary for the implementation of a rigorous BBA. To this aim it is necessary to have the same input used for the first processing (IPs, GCP coordinates and image coordinates of the TPs), but also the approximate EPs and the approximate ground coordinates of each TP (Figure 3).

GNSS positions, when available, represent a strong constraint for the estimation of the camera poses, because they are known with a few centimeters of error. In order to use them as pseudo-observations it is necessary to estimate the lever-arm between the camera and the GNSS antenna (*i.e.*, the components of the 3D vector that connect the phase center of the GNSS antenna and the camera projection center, expressed in the camera reference system). The calibration vector can be estimated using the images acquired during the survey. However, it is better to solve this task with a proper geometric calibration phase executed before the survey. This consists in the acquisition of a series of images of a 3D calibration polygon, storing also the GNSS antenna positions at the time of each image acquisition. The calibration polygon has to be an object with a size similar to the buildings that will be framed during the survey. It has to be accessible, characterized by high chromatic contrast and by the presence of evident details, located on different planes, such as a building façade with balconies. A low number of GCPs, known with few millimeters of error, have to be measured on the building façade to scale and rotate the photogrammetric problem.

The equation of the geometric calibration is:

(3) $$\Delta X_{camera\_ref}=R_{\omega }R_{\phi }R_{\kappa }(X_{cam}-X_{GNSS})$$

where ∆*X_camera_ref_* are the components of the lever-arm expressed in the camera reference system, *R_ω_R_ϕ_R_κ_* are the rotation matrices about the gimbal angles, *X_cam_* are the coordinates of the camera projection centers and *X_GNSS_* are the coordinates of the GNSS antenna.

As stressed before, the second BBA is computed rigorously implementing the linearized collinearity equations, through the use of a non-linear least square adjustment (computed using the output of the first BBA as approximated values). During this second computation also the residual correction of the IPs are estimated in order to have a more robust final solution.

## 3. Test

**Figure 4:**
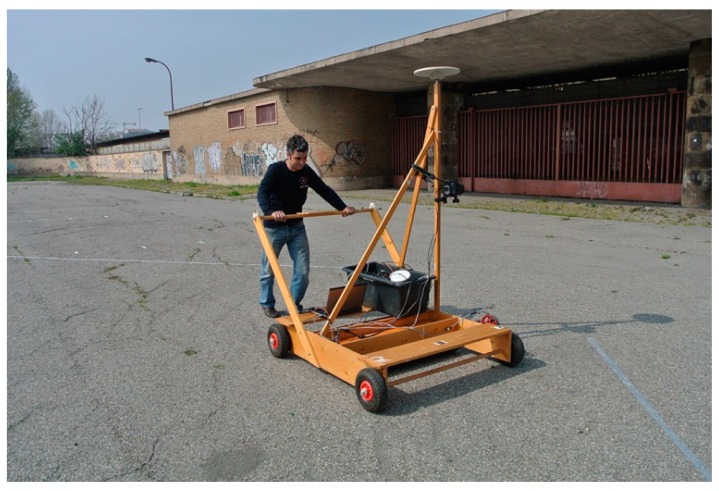
The system during the survey: the (orange) GPR is located at the bottom of the wood carrier, the GPS antenna is on the top of the stake, while the camera is fixed below it. The box in the lower part contains the other components of the system: the batteries and the hardware for controlling the camera. The laptop is near it. In the background it is possible to see the framed building. The façade is made of regular bricks, but some graffiti helps diversifying a bit the texture. This is not true for the gate. Note the different lighting conditions between the wall and the gate.

The test was performed in an area of Milan (Italy), where some buildings are present. The façades are regular (Figure 4), which is quite frequent in urban architecture but detrimental for photogrammetric positioning. Furthermore, in the chosen site, the buildings are quite low (approximately 2.5 m high), thus leaving the upper part of some photos without usable tie points. The buildings are located only to the North of the test area, thus preserving a good satellite visibility (over Italy, because of the orbital inclination, GPS satellites are never visible for −30° < α < +30°, where α is the azimuth,). This allows obtaining an accurate GPS solution to be used as a reference one. To obtain cm-level accuracy with phase double differential positioning, a geodetic L1/L2 GPS was used, together with a master station of the same kind. A Nikon D70s camera has been used. A 20 mm fixed focal length has been chosen, thus minimizing the lens distortion errors and guaranteeing an optimal calibration of the camera itself. Moreover, a wide-angle lens with short focal length has been used, allowing focusing already at a distance of few meters, as expected between a vehicle and side buildings. Image acquisition has been triggered by a laptop, equipped with software and external hardware specifically realized for shooting at regular intervals. This electronic device sends a command to the Nikon USB port and receives a square wave from the camera flash contact, which is considered synchronous with the shutter opening. At this time, the software records the photo code and the PC time in a text file. The error is smaller than 0.001 s.

The GPR device used in this test has no internal clock and the acquisition is triggered by an odometer signal (so it is not regular in time, but in space). A timestamp is associated to the GPR measures when a NMEA message is received from a GNSS device. Because the proposed approach must work even without GNSS, a fictitious NMEA message containing the PC time must be generated and sent to the GPR: this is performed by an *ad hoc* designed software. Since the NMEA message is not synchronous to the GPR acquisition, it has to be generated at least at 100 Hz in order to have a negligible error (this means less than 0.02 m error along-track). The GNSS positions, instead, refer to the GPS timescale, with errors on the order of 50 nanoseconds.

As a result of all this the GPR measurements and the positions obtained by the different sensors have different sampling intervals and clocks. In order to integrate them, a common timescale is needed. Since the proposed approach must work even without GNSS, the GPS time cannot be used as the common timescale. As a second option, one can consider the time of the laptop used for storing the data of the different sensors because this is always available and its stability is acceptable for the duration of a single strip in the test that was carried out. Laptop timescale cannot give an absolute time: however, this is generally not required. Thus it was decided to use the laptop as a common timescale to which refer the observations collected by the sensors (GPR, GNSS and camera). This means that, when GNSS positions are available, the synchronization between laptop and GPS time is required. To this aim, a simple software able to read/store simultaneous PC and GPS timestamps and to estimate a linear transformation between the two timescales has been realized. This is indeed a suboptimal solution, because the laptop clock drift and instability can have a relevant impact even after ten minutes which is however a time span larger than the one required for acquiring a single GPR strip. As a matter of facts, the maximum error obtained from numerous tests was in the order of 0.02 s (less than 0.04 m error). The laptop clock stability is also sufficient for the position interpolation to the GPR acquisition epochs that was performed by cubic spline functions. 

The instruments were installed on a wood carrier (Figure 4): the GPR was placed in the bottom part, while a GPS Trimble Zephyr Geodetic antenna was screwed on a stake of the carrier, and the camera was fixed to the stake itself, below the antenna. The carrier supported the GPS receiver (Trimble 5700) with its battery, and a laptop running the software and operating the camera.

The GPR survey was performed simply by pushing the carrier by hand, a usual approach for GPR surveys in small areas. In this way, 10 strips parallel to the building were realized. The on-board GPS gathered raw data at 1 Hz rate, while the camera acquired images every 2–3 s. During the survey, the lighting conditions often changed because of the frequent variations of the cloudiness, so the collected images show relevant differences.

**Figure 5:**
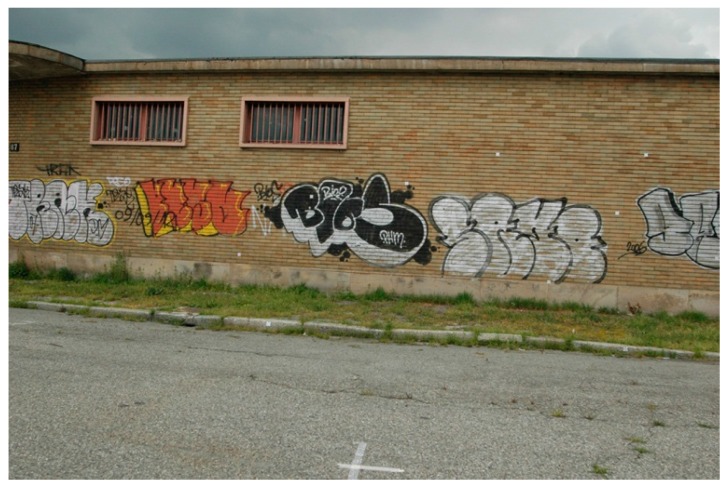
Example of image used for the geometric calibration. The (white) targets are visible on the wall and on the rise of the sidewalk.

To perform the geometric calibration, at the end of the survey some markers placed on the façade were shot on an arc-shaped trajectory. Twelve targets were placed on the façade and their coordinates were accurately estimated via topographical survey (with an error of few millimeters). The collected images (Figure 5), together with simultaneous GPS positions, allow determining the lever arm between GPS and camera. For obtaining an accurate solution, this operation is, at the moment, required for every survey, because the camera is not fixed in a reproducible position of the stake. Otherwise, it would be sufficient a “once-for-all” geometric calibration of the system.

The GCPs coordinates are to be extracted from the Milan urban map (scale 1:1000). Nevertheless, in this small area, the map showed a low accuracy, simplifying too much the building for our purposes. In fact, as required by the usual cartographic generalization, the roofing was partially cut, while the rounded edges (visible in Figure 4) were represented as sharp edges, so only two points at the lower left and lower right sides of the main building could be extracted from the map. They are clearly not sufficient to constrain the degrees of freedom of the photogrammetric inverse problem. Therefore, the map was not used and 10 points were surveyed with a Leica Geosystems total station, achieving a few millimeters accuracy. The instrument was placed on stations points whose coordinates were measured with a static GPS survey (20’ data acquisition).

This allowed roto-translating the topographical results into the global reference frame (European Terrestrial Reference Frame 1989, ETRF89) which was used in the whole test. The topographical survey took just one hour and a half and could have been performed simultaneously to the GPR survey.

The raw GPS data of the whole test were post-processed with Trimble commercial software. The GNSS permanent station located nearly 2300 m away from the test site was considered as the master station. The International GNSS Service (IGS) precise ephemerides, the National Geodetic Survey (NGS) antenna calibration parameters, and the Niell tropospheric model were used in the computations. A mask angle of 15° was set: during the test, the number of used satellites was never less than 8, while PDOP was never greater than 2. Double difference solutions, with phase ambiguities fixed as an integer, were obtained at all epochs, and the standard deviation (σ) was never greater than 0.02 m. The GPS solutions have been interpolated to the shot instants for comparison with the photogrammetric solutions.

The photogrammetric block is made by 301 images, divided into 10 strips. In this test, several critical aspects causing possible errors in the photogrammetric solution were present: the GCPs and the tie points were all almost coplanar, the baselines between subsequent images are very short, and the observed scene shows repetitive textures, particularly in correspondence of the gate. However the adopted multi-image approach allowed efficiently overcoming such problems thus proving the robustness of the method. The tie points have been extracted with commercial software Agisoft Photoscan, in a completely automated way, and then filtered according the previously described approach (§2). 16000 tie points resulted, corresponding to about 2650 object points. On the images the average number of tie points is 56, with a maximum of 90, while the average multiplicity is equal to 6, with maximum 77. The average overlapping of images is 82%. The first bundle block adjustment was performed with the commercial software Photomodeler, while the second one was done with the scientific software Calge [32]. The GCPs, obtained by topographical survey, has been approximated as fixed points, *i.e.*, with infinite accuracy.

Three different scenarios have been considered:

(a) a purely photogrammetric solution assuming that GPS signal is unavailable;
(b) a photogrammetric solution with GPS positions, available at the beginning and at the end of each strip, that can be used for constraining the camera projection centers;
(c) a photogrammetric solution with few GPS positions randomly distributed along some of the strips and at the beginning and at the end of each strip.

## 4. Results

The root mean square (RMS) of the standard deviations of the estimated coordinates of the camera projection centers, obtained by the final bundle block adjustment in the three scenarios, are presented in Table 1. Those nominal errors are very small, being always less than 0.01 m. The use of some GPS observations does not significantly influence the value of this parameter.

**Table 1:** RMS of standard deviations of the coordinates of the camera projection centers.

	Scenario a	Scenario b	Scenario c
East (m)	0.006	0.007	0.005
North (m)	0.005	0.005	0.004
height (m)	0.009	0.008	0.006

A more accurate analysis of the true performance could be achieved considering the differences between the estimated coordinates and the GPS reference solutions at corresponding epochs. The largest errors (Table 2) are in the East-West direction, approximately orthogonal to the camera orientation, and they are on the order of 0.1 m. The results show a significant improvement when some GPS pseudo-observations are added at the beginning and at the end of each strip. Further improvements have been reached adding also some GPS spotted pseudo-observations.

**Table 2:** RMSe of the differences between the photogrammetric solutions and the GPS positions.

	Scenario a	Scenario b	Scenario c
East (m)	0.137	0.081	0.035
North (m)	0.121	0.054	0.021
height (m)	0.099	0.024	0.014

**Figure 6:**
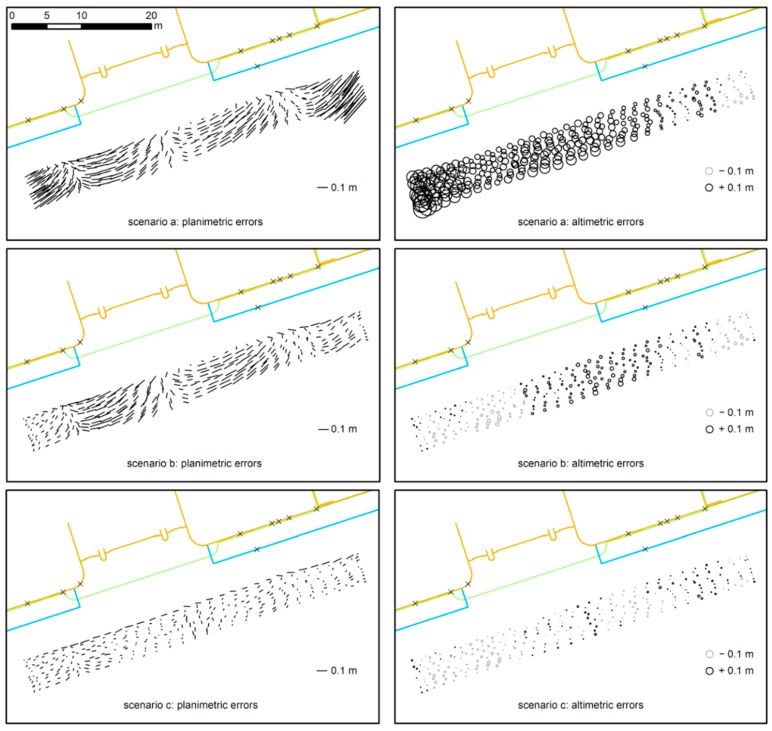
Planimetric and altimetric differences between the photogrammetric and the GPS solutions for the different scenarios. In the upper part the (true) plan of the buildings, the gate (orange), the roofing (green) and the sidewalk (blue) are visible. GCPs are represented by crosses.

When analyzing the errors along the tracks (see Figure 6), the effect of the introduction of the GPS positions is more evident. Clear improvements are already visible when few GPS solutions are added at the beginning and at the end of each strip, as constraint of the camera projection centers. 

When GPS is not available (scenario a), the accuracy is dependent on the block conformation and the distribution of the GCPs on the framed objects. In fact, the higher planimetric differences are located at the beginning and at the end of the strips, where the baselines are shorter because the carrier moved slowly. Moreover, a correlation with the characteristics of the building surface is visible. The absence of GCPs at the gate position (in the central part of the block) is the main cause of the systematic behavior of planimetric errors in that area. The altimetric errors are mainly caused by the fact that GCPs are visible only in a quite small horizontal band of each image which implies a less robust estimation of the roll angle (camera is pointing towards the building façade). This effect is smaller in the eastern part of the photogrammetric block because of the GCPs on the sidewalk.

## 5. Conclusions

The paper proved that GPR positioning in GNSS-challenged environments could be affordably performed using a vision-based system. Moreover, the accuracy of 0.2–0.3 m, required for the 3D modeling of underground detected objects, could be only achieved for the whole trajectory by using very accurate GCPs or/and by constraining the camera perspective centers with some GPS positions. In this paper, a photogrammetric approach matching the requirements for GPR positioning is presented, together with the results of a test performed in an area where vision-based methods could have poor performances. Results demonstrated that the solution was robust enough for recovering the trajectory even in these critical situations, such as poor-textured framed surfaces, short baselines, remarkable lighting variations and low intersection angles. Moreover, the test was performed adopting a suboptimal synchronization solution. Nevertheless, the differences with respect to the reference GPS solution were never greater than 0.35 m. Such performance could be generally achieved just with more expensive instrumentations, like terrestrial LIDAR or high-level INSs. 

Future development of the presented work will be the creation of an *ad hoc* software able to perform the whole processing chain, from the tie points extraction to the GPR georeferencing. This will allow to be independent from commercial solutions, and also to speed up the procedure, automatically optimize some parameters, decrease the manual operations for tie points selection, avoid the format conversions and so on. Moreover, in future works, the GPR solutions will be compared with the true positions of the buried objects, with the aim to investigate the underground error propagation.
